# *In vitro* assessment of anti-proliferative effect induced by *α*-mangostin from *Cratoxylum arborescens* on HeLa cells

**DOI:** 10.7717/peerj.3460

**Published:** 2017-07-21

**Authors:** Aisha I. El habbash, Najihah Mohd Hashim, Mohamed Yousif Ibrahim, Maizatulakmal Yahayu, Fatima Abd Elmutaal Omer, Mashitoh Abd Rahman, Noraziah Nordin, Gwendoline Ee Cheng Lian

**Affiliations:** 1 Pharmacy Department, Faculty of Medicine, University of Malaya, Kuala Lumpur, Malaysia; 2 Center for Natural Products and Drug Discovery (CENAR), University of Malaya, Kuala Lumpur, Malaysia; 3 Department of Bioproduct Research & Innovation, Institute of Bioproduct Development (IBD), Universiti Teknologi Malaysia, Johor Bahru, Malaysia; 4 Medical Science 1, Faculty of Medicine and Health Sciences, Universiti Sains Islam, Malaysia, Kuala Lumpur, Malaysia; 5 Department of Chemistry, Faculty of Science, Universiti Putra Malaysia, Selangor, Malaysia

**Keywords:** Antitumor, α-mangostin, Apoptosis, HeLa

## Abstract

Natural medicinal products possess diverse chemical structures and have been an essential source for drug discovery. Therefore, in this study, α-mangostin (AM) is a plant-derived compound was investigated for the apoptotic effect on human cervical cancer cells (HeLa). The cytotoxic effects of AM on the viability of HeLa and human normal ovarian cell line (SV40) were evaluated by using MTT assay. Results showed that AM inhibited HeLa cells viability at concentration- and time-dependent manner with IC_50_ value of 24.53 ± 1.48 µM at 24 h. The apoptogenic effects of AM on HeLa were assessed using fluorescence microscopy analysis. The effect of AM on cell proliferation was also studied through clonogenic assay. ROS production evaluation, flow cytometry (cell cycle) analysis, caspases 3/7, 8, and 9 assessment and multiple cytotoxicity assays were conducted to determine the mechanism of cell apoptosis. This was associated with G2/M phase cell cycle arrest and elevation in ROS production. AM induced mitochondrial apoptosis which was confirmed based on the significant increase in the levels of caspases 3/7 and 9 in a dose-dependent manner. Furthermore, the MMP disruption and increased cell permeability, concurrent with cytochrome c release from the mitochondria to the cytosol provided evidence that AM can induce apoptosis via mitochondrial-dependent pathway. AM exerted a remarkable antitumor effect and induced characteristic apoptogenic morphological changes on HeLa cells, which indicates the occurrence of cell death. This study reveals that AM could be a potential antitumor compound on cervical cancer *in vitro* and can be considered for further cervical cancer preclinical and *in vivo* testing.

## Introduction

**Figure 1:**
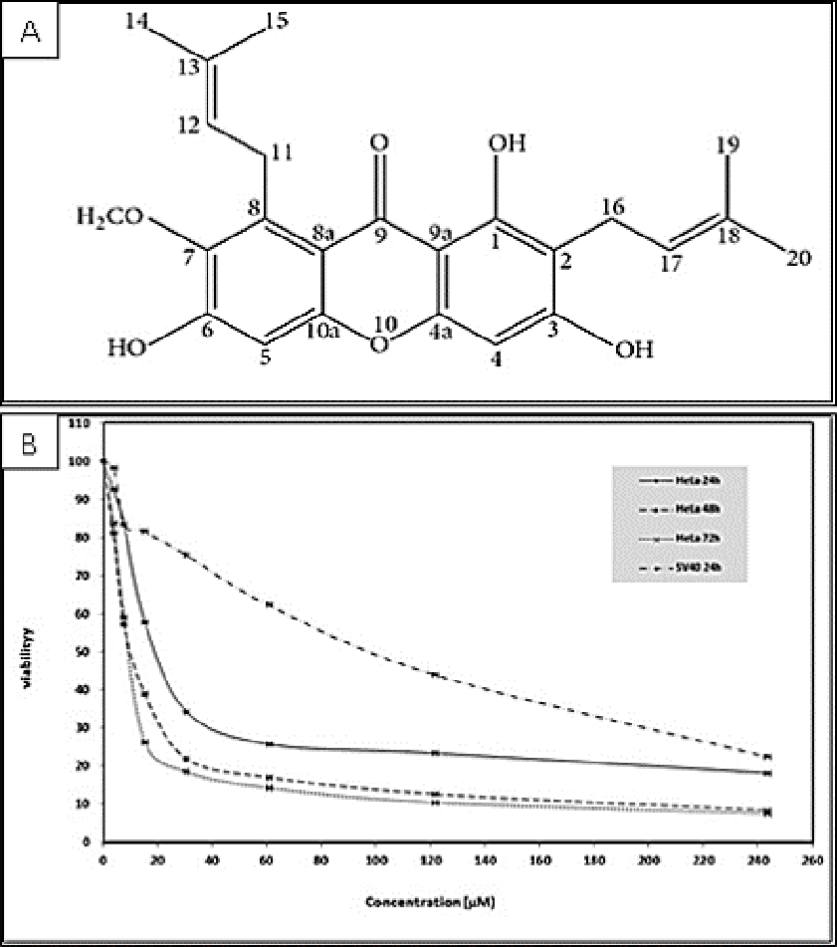
(A) Chemical structure of α-mangostin (AM) isolated from *C. arborescens* (Sidahmed et al., 2013). (B) Toxic effects of AM on HeLa and SV40 cells viability. The viability of HeLa cells was measured after 24, 48, and 72 h of treatment. The viability of SV40 cells was measured after 24 h of treatment. Each point expresses the mean ± S.D. of three separated experiments.

Various tropical plants exhibit interesting biological activities for therapeutic applications; several new biologically active compounds may exert a synergetic anticarcinogenic effect when used with standard drug treatments (Pedraza-Chaverri et al., 2008). *Cratoxylum arborescens* (Blume), a well-known Asian herbal medicine, belongs to the Guttiferae family. The leaves, bark, and root of this plant are traditionally used to treat fever, ulcers, coughs, itchiness, diarrhea, and abdominal disorders (Sidahmed et al., 2013). The main phytochemical compounds found in *C. arborescens* are xanthones, which exhibit various significant pharmacological properties (Sidahmed et al., 2013). Xanthones are chemopreventive and therapeutic agents and can effectively inhibit tumor initiation and progression (Matsumoto et al., 2005; Pedro et al., 2002). The biological activities of xanthones are associated with their tricyclic scaffold but vary according to the type and/or position of the varied substituents (Wong et al., 2013). AM is a yellow powder with a xanthone core structure (Fig. 1A) and is one of the major secondary metabolite of xanthones; this compound exhibits a wide spectrum of biological activities as an analgesic, anti-HIV agent, and immunity booster (Abdullah, Al-Kubaisy & Mokhtar, 2013). AM also acts as an antiparasitic, antidiabetic (Ibrahim et al., 2014b), anti-inflammatory (Chairungsrilerd et al., 1996), antioxidant (Márquez-Valadez et al., 2009), anti-tumor (Chitchumroonchokchai et al., 2013), antibacterial (Negi, Jayaprakasha & Jena, 2008; Sakagami et al., 2005), antifungal (Pedraza-Chaverri et al., 2008), cardio protective (Devi Sampath & Vijayaraghavan, 2007), anti-ulcer (Sidahmed et al., 2013) and can also act as well as an anti-obesity agent (Ibrahim et al., 2015). As an anti-cancer agent, AM has been reported to induce apoptosis and cell death in different types of cancer cells (Ibrahim et al., 2014b). AM induces apoptosis and cell cycle arrest in human colon cancer DLD-1 cells (Matsumoto et al., 2005), apoptosis in human breast cancer MCF-7 cells with regulation of NF-κB and Hsp70 protein modulation (Ibrahim et al., 2014b), apoptosis in human breast cancer MDA-MB-231 cells by NF-κB and HSP70 signaling pathways (Ibrahim et al., 2014a), and mitochondrial dysfunction in human leukemia HL60 cells (Matsumoto et al., 2004) . Thus far, significant cytotoxic effect of AM has not been observed in cervical cancer cells; thus, this study investigated the antitumor effect of this compound on cervical cancer cell line HeLa.

## Materials and Methods

### Extraction and isolation of AM from *C. arborescens*

AM was extracted from the stem bark of *C. arborescens*, wild trees growing in Malaysia. A voucher specimen was deposited at the Herbarium, Department of Biology, University Putra Malaysia. Hexane, chloroform and methanol extracts were collected. The characterization and identification of AM have been done using JOEL ECA-400 spectrometer and mass spectra data was recorded using a Shimadzu GCMS-QP5050 spectrometer (Sidahmed et al., 2013). The purity of AM was confirmed to be 100% by HPLC-LC 20 AD (Shimadzu, Japan) using reversed-phase C18, 2.5 mm column from Waters with 10% acetonitrile in water. One peak has been observed between 7–7.5 min. The detection of the peak was at 245 nm wavelength using a Gilson absorbance detector (UV–VIS165 Gilson USA).

### Cell culture and reagents

The human cervical cell line (HeLa), and human normal ovarian cell line (SV40) were obtained from the American Type Culture Collection (ATCC). Cells were maintained in RPMI media, supplemented with 10% fetal bovine serum, 100 U/ml penicillin, and 0.1 mg/ml streptomycin. The cells were then incubated at 37 °C with 5% CO2 saturation. Cells were used in the linear phase of growth with a passage number of 20 or less. Phosphate buffered saline (PBS), dimethyl sulfoxide (DMSO), 3-(4, 5-dimethylthiazol-2-yl)-2, 5-diphenyltetrazoliumbromide (MTT), and other reagents were acquired from Sigma Chemical Co., St. Louis, MO, USA.

### Treatment preparation

Stock solution of AM (2.43 mM) was prepared in dimethyl sulfoxide (DMSO), filter sterilized before use and diluted with the prepared media to the selected treatment concentrations.

### Viability assay

The cytotoxic effect of AM on cervical cancer cells (HeLa) and normal ovarian cells (SV40) were evaluated by MTT assay (Mosmann, 1983). The cells were seeded at 1 × 10^4^ cells per well in a 96-well plate and incubated at 37 °C overnight. Cells were treated with increasing doses of AM by 2-fold dilution starting with 121.8 µM until 1.90 µM. Cells were treated for 24, 48, and 72 h, then procedures were conducted as prescribed in details by Ibrahim et al. (2014b). The MTT assay results were obtained using an ELISA plate reader (Tecan Group Ltd., Mannedorf, Switzerland) at 570 nm. The MTT assay results depend on the formation of purple formazan crystals from the reduction of tetrazolium salt by dehydrogenase enzymes in metabolically active cells. This experiment was conducted to determine the half-maximal inhibitory concentration (IC_50_) values of AM against HeLa cells. All experiments were performed in triplicate to evaluate half-maximal inhibitory concentration for AM against the HeLa cell line. The IC_50_ (the concentration of AM necessary to produce 50% inhibition of cell growth) was calculated from the following nonlinear equation of the survival fraction curve:

\begin{eqnarray*}Y=a{X}^{3}+b{X}^{2}+cX+d \end{eqnarray*}

where:

\begin{eqnarray*}& & Y=(\text{the surviving fraction when there is a 50% inhibition of cell growth}). \end{eqnarray*}

\begin{eqnarray*}& & X=\text{dose of AM induces 50% inhibition}. \end{eqnarray*}

\begin{eqnarray*}& & a,b,c~\text{and}~d=\text{constant values}. \end{eqnarray*}

### Proliferation assay

HeLa (400 cells/plate) cells were plated in small flasks and incubated with 5% CO2 overnight. The incubated cells were treated with gradually increasing three doses of AM (IC_50_, IC_50_  ±  12 µM). Cells were treated with (12, 24, and 36 µM) for 24 h. The cells were then washed, and fresh medium were added. After 14 days of incubation, surviving colonies were first fixed with ethanol 70% (v/v) for 10 min and then stained with crystal violet (Coomassie Blue Stain). After staining, colonies containing more than 50 cells were counted. Plating efficiency (PE) was evaluated, and the fraction of surviving cells at a given treatment was calculated.

\begin{eqnarray*}& & \text{Plating Efficiency (PE)}=(\text{No. of colonies counted/No. of cells plated})\times 100 \end{eqnarray*}

\begin{eqnarray*}& & \text{Survival Fraction}=(\text{PE of treated sample/PE of control})\times 100. \end{eqnarray*}

### Assessment of apoptotic morphological changes in cells using propidium iodide and acridine orange double staining (AO/PI)

The morphological changes in apoptotic HeLa cells were evaluated using AO/PI double staining and visualized by fluorescence microscopy (Leica with Q-Floro Software, Leica Microsystems). Cells were seeded at a concentration of 5 × 10^5^ cells/mL in a 25 mL culture flask, incubated overnight at 37 °C, and then treated with the IC_50_ concentration of AM. The cells were treated for 24, 48, and 72 h. 10 µL of two fluorescent dyes, namely, acridine orange (AO, 10 µg/mL) and propidium iodide (PI, 10 µg/mL) were added to the cells. The rest of procedures were conducted as mentioned in details by Ibrahim et al. (2014a). The morphological principles used for the classification of viable, apoptotic, and necrotic cells were mentioned in details by Rahman et al. (2016).

### Hoechst 33258

The morphological analysis of (HeLa) cell apoptosis was confirmed by nuclear staining with Hoechst 33258 dye. The cells were seeded at a concentration of 2 × 10^5^ in a 25 mL culture flask for 24 h to allow attachment. The cells were then treated with IC_50_ concentration of AM and incubated for 24, 48, and 72 h. After removal of the culture medium, the cells were fixed with ethanol 70% (v/v) for 10 min. The cells were then washed twice with PBS and stained with Hoechst 33258 dye (1 µg/mL) for 15 min in an incubator with 5% CO_2_. The samples were detected by fluorescence microscopy. Apoptotic cells were identified according to the characteristic nuclear morphological changes in the cells, including reduction in volume and chromatin condensation.

### Cell cycle analysis

Cell cycle kinetics was evaluated by flow cytometry analysis using DNA labeling with PI by using a previously described method with minor modifications (Nicoletti et al., 1991). HeLa cells were seeded at a concentration of 2 × 10^5^ cells/mL in a 25 mL culture flask, incubated overnight at 37 °C, and treated with IC_50_ concentration of AM for 24, 48, and 72 h. After the treatment, the cells were centrifuged at 1,000 rpm for 10 min. The supernatant was removed, and the pellet was washed with warm PBS twice to discard the remaining media. To preserve the cell structure, the cells were fixed by mixing with 700 µL of 90% pre-cooled ethanol overnight at 4 °C. The cells were then centrifuged at 250 *g* for 10 min, and ethanol was completely discarded. After washing with PBS, the cell pellets were resuspended in PBS (700 µL) and RNase (25 µL, 10 mg/mL). The PI dye (50 µL, 1 mg/mL) was added, and the cells were retained for 30 min at 37 °C. DNA contents of the cells were then analyzed by flow cytometry.

### Caspase 3/7, 8 and 9 assays

The activities of caspases 3/7, 8, and 9 were determined by colorimetric-based assays (Caspase 3/7 assay, R&D Systems Kit; Caspase 8, R&D Systems Kit; and Caspase 9 assay, R&D Systems Kit) in a time-dependent study. HeLa cells were seeded and incubated for 24 h at 37 °C. The cells were treated with IC_50_ of AM for 24, 48, and 72 h and collected by centrifugation at 250 g for 10 min. The supernatant was eliminated, and 25 µL of cold protein lysis buffer was added per 1 ×10^6^ cells. The cell lysate was incubated in ice for 10 min and centrifuged at 10,000 *g* for 1 min. The supernatant was retained, and about 50 µL of the supernatant (protein) was transferred into a well of 96-well plates in triplicate. The well was then added with 50 µL of reaction buffer and 5 µL of caspase and incubated for 1–2 h at 37 °C. The plates were read using the ELISA microplate reader at a wavelength of 405 nm.

### DCFH-DA Cellular Reactive Oxygen Species (ROS) detection assay

Intracellular reactive oxygen species (ROS) production levels in HeLa cells were calculated using 2′, 7′-dichlorofluorescin diacetate (DCFH-DA) in a concentration dependent study. Approximately 5 × 10^3^ cells per well were seeded in a 96-well black plate and incubated at 37 °C overnight. The cells were then treated with gradually increasing concentrations of AM (12, 24, and 36 µM). The assay was then conducted as prescribed by Ibrahim et al. (2014b).

### Multiple cytotoxicity assay

Multiple cytotoxicity assays were conducted using the Cellomics^®^ Multiparameter Cytotoxicity 3 Kit (Thermo Scientific, Pittsburgh, PA, USA) as mentioned in details by Cheah et al. (2011). HeLa cells were analyzed using the Array Scan HCS system after 24, 48, and 72 h of treatment with IC_50_ concentration of AM (24 µM).

### Statistical analysis

Each experiment was performed at least three times. Results were expressed as the means value ± standard deviation (SD). Statistical analysis was performed with Microsoft Excel and one-way analysis of variance (ANOVA) with Tukey Multiple Comparison Test to compare all columns vs. control using Graph Pad Prism software (version 4.0; Graph Pad Software Inc., San Diego, CA). Statistical significance is expressed as ^∗^*p* < 0.05 or lower.

## Results

### AM inhibited HeLa cells *in vitro*

The cytotoxicity of AM was determined by MTT assay. The cell lines used in this study included human cervical adenocarcinoma cell (HeLa), human epidermoid carcinoma cell (Ca Ski) and normal human epithelial ovarian cell (SV40). The MTT assay results indicated that AM treatment markedly decreased the cell viability of the treated HeLa and Ca Ski cells compared with that of cells unexposed to AM. The IC_50_ values of AM for HeLa and Ca Ski cells were 24.54 ± 1.5 µM and 51.73 ±  2.87 µM, respectively, after 24 h as shown in Table 1, HeLa cells exhibited higher sensitivity to AM and obtained lower IC_50_ values than Ca Ski cells. The IC_50_ values of AM for HeLa cells at the studied durations of 24, 48, and 72 h are shown in Fig. 1. The viability percentage decreased significantly from 24 to 48 h but was not significantly different between 48 and 72 h in HeLa cells exposed to AM. Fortunately, AM exerted lower toxicity on SV40 compared with that on HeLa and Ca Ski cells, and exhibited an IC_50_ value of 93.95  ±  5.11 µM as shown in Table 1. These findings demonstrated that AM inhibits the viability of cervical cancer cell line HeLa.

**Table 1:** The IC_50_ values AM against the viability of HeLa, Ca Ski and SV40 cells.

Cell line	HeLa	Ca Ski	SV40
IC_50_ at 24 h (µM)	24.54 ± 1.5	51.73 ± 2.87	93.95 ± 5.11

### AM significantly inhibited cell proliferation and colony formation of HeLa cells

**Figure 2:**
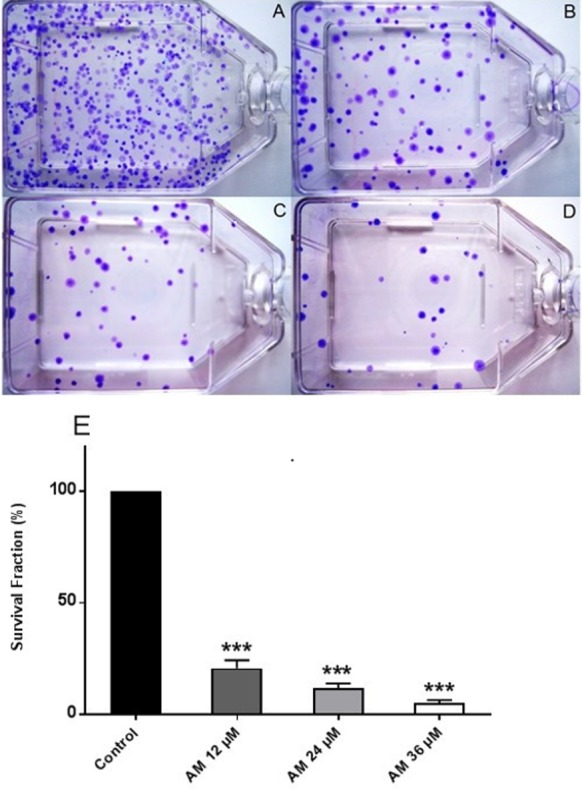
Effects of AM on HeLa cell colony formation as measured by Clonogenic assay. Cells were treated with gradually increased concentrations of AM for 24 h and then cultured for 14 days. (A) HeLa control, (B, C, D) HeLa cells treated with (12, 24, and 36 µM) of AM respectively, (E) Effect of gradually increased concentration of AM on colony formation capability of HeLa cells, values as mean ± S.D, (^∗∗∗^*P* < 0.001) vs. control.

Healthy and active cells can reproduce and form colonies. Anti-cancer drugs can lead to loss of reproductive integrity and proliferation of cancer cells. Plating efficiency is the percentage of cells that can grow into colonies after treatment, whereas survival fraction is the percentage of cells that can reproduce after treatment. The effects of AM on the colony formation efficiency of HeLa cells were determined. The results indicated that HeLa can form a large number of small colonies (Fig. 2A). Obviously, 24 h of AM treatment caused a dose-dependent inhibition of colony formation on HeLa cells. This inhibitory effect was observed from the lowest AM concentration and proportionally increased with increasing doses of AM (Fig. 2E). All the tested fractions of AM significantly inhibited cell proliferation and colony formation of HeLa cells compared with the control. These findings suggested that HeLa cells cannot actively reproduce and form colonies when treated with tested fractions of AM.

### Determination of apoptotic morphological changes in HeLa cells by using propidium iodide and acridine orange double staining (AO/PI)

**Figure 3:**
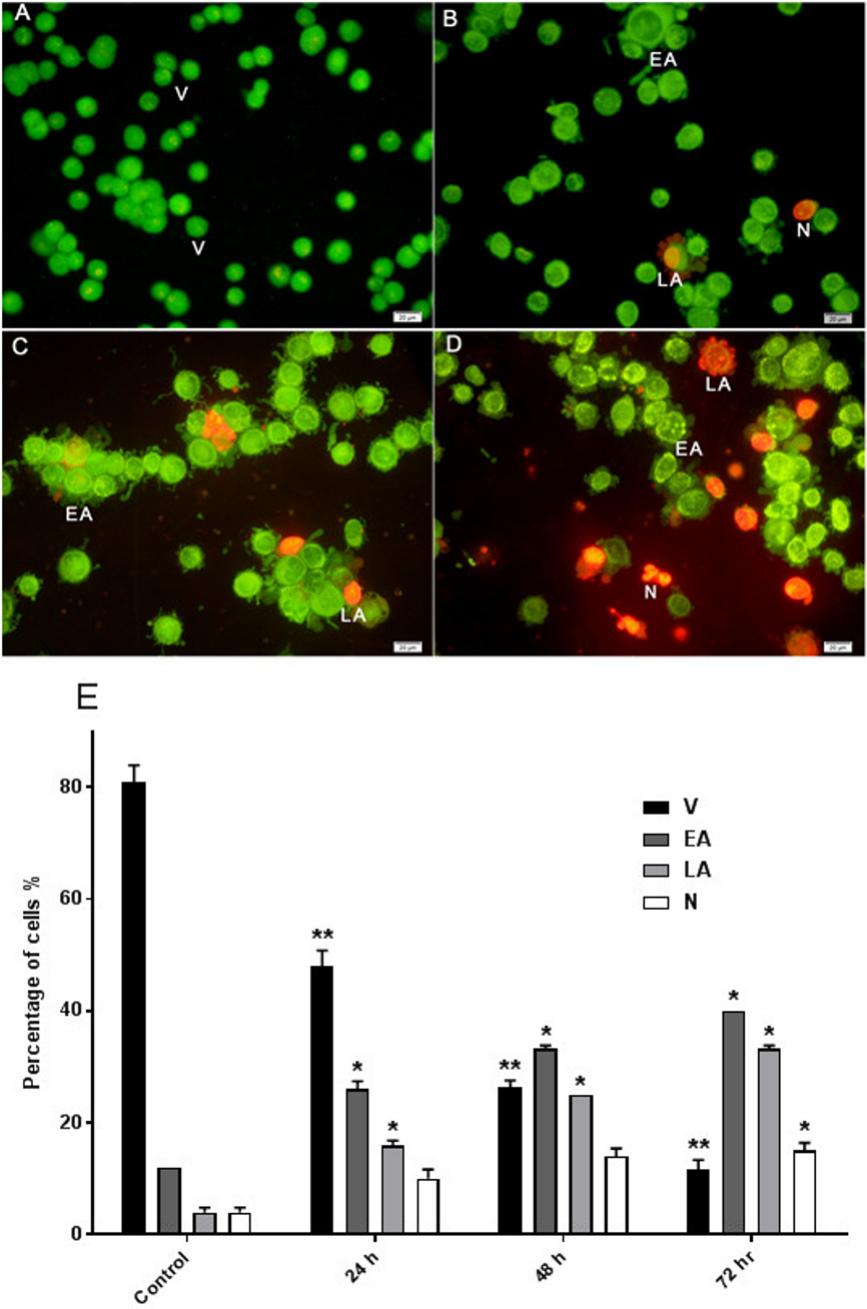
Effects of AM on HeLa cells as measured by Acridine Orange and Propidium Iodide double-staining in a time dependant manner. Viable (A), early apoptotic (B), late apoptotic (C) and secondary necrotic (D) features were seen. Note: V: viable, EA: early apoptosis, LA late apoptosis and N: necrosis. (E) Percentages of viable, early apoptotic, late apoptosis and necrotic cells after AM treatment. The statistical significance is expressed as ^∗^*P* < 0.05; ^∗∗^*P* < 0.01.

The effect of AM on HeLa cells was determined with AO/PI double staining by using a fluorescence microscope. The toxicity of AM causes a number of morphological changes, indicating its apoptotic effect in a time-dependent manner. At 24 h, the cells exhibited early apoptosis, as evidenced by the bright green fluorescence caused by AO binding to the fragmented DNA (Fig. 3B). By contrast, control cells (untreated) showed green normal nuclear structures (Fig. 3A). After AM treatment for 48 h, moderate apoptotic features, such as cell blebbing and nuclear chromatin condensation, were recognized (Fig. 3C). Moreover, cells treated with AM for 72 h exhibited features of late apoptosis and showed reddish-orange fluorescence caused by AO intervention within the denatured DNA (Fig. 3D). In addition, numbers of viable cells decreased significantly after exposure to AM, as well as numbers of apoptotic cells increased proportionally with treatment duration (Fig. 3E). These findings demonstrated that AM exerts an apoptogenic effect on HeLa cells in a time-dependent manner.

### Nuclear changes and apoptotic features by Hoechst 33258 staining

**Figure 4:**
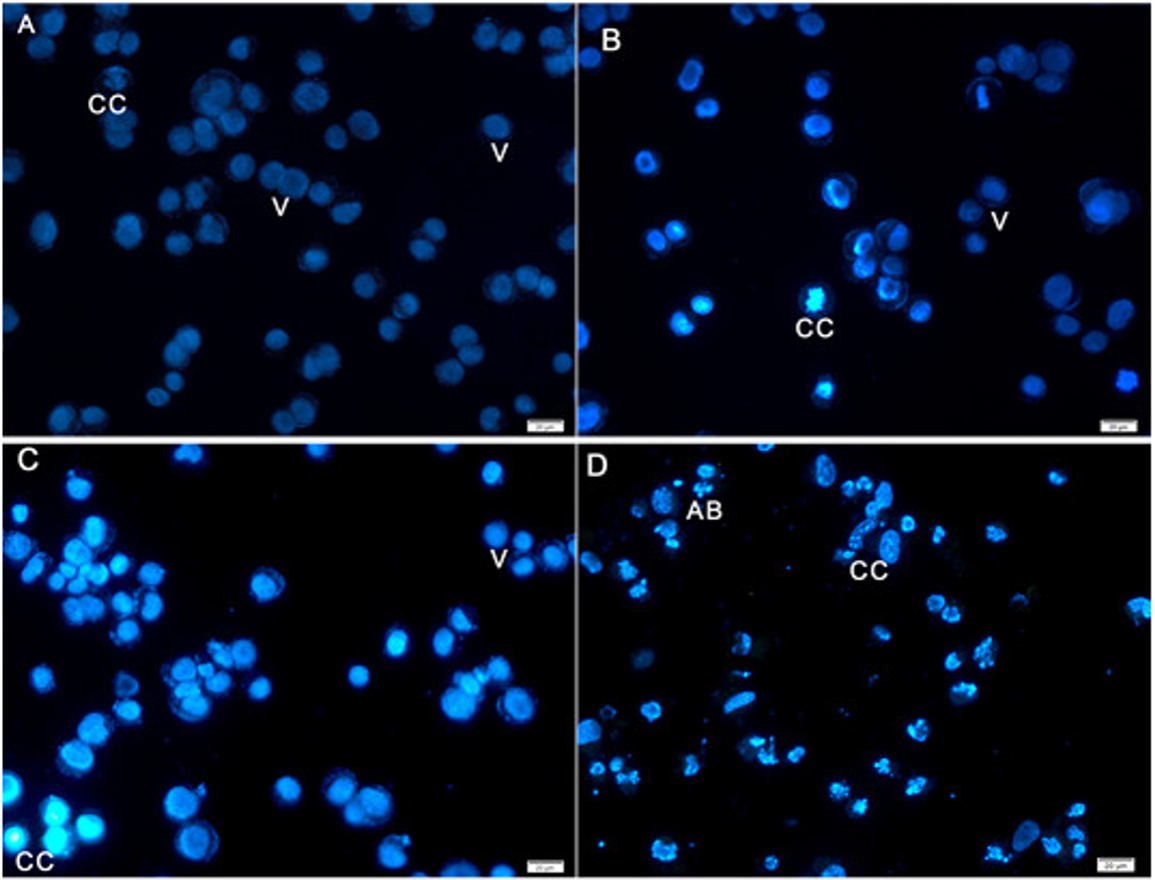
Fluorescent micrograph of Hoechst 33258 dye stained HeLa cells. (A) HeLa control, (B, C, D) HeLa treated with AM (24 µM) for 24, 48, and 72 h respectively. Note, V: viable, CC: chromatin condensation, and AB: Apoptotic bodies.

Hoechst 33258 staining results revealed classic apoptotic hallmarks, such as nuclear chromatin condensation, DNA fragmentation, plasma membrane blebbing, and formation of apoptotic bodies, followed by removal of cells by phagocytes. The morphological changes in HeLa cells caused by apoptosis were examined using cell-permeable DNA dye Hoechst 33258 through fluorescence microscopy analysis. The normal untreated control cells showed normal intact nuclei with faint staining (Fig. 4A). The cells treated for 24 h showed fragmented nuclei and nuclear blebbing, which are indicative of apoptosis (Fig. 4B). Moreover, cells treated with AM for 48 h showed numerous apoptotic cells with condensed, fragmented nucleus and a much brighter color (Fig. 4C). Furthermore, cells treated with AM for 72 h exhibited marked characteristic features of apoptosis, including marked nuclear condensation, intense brightness, and formation of dense, apoptotic bodies (Fig. 4D). These results indicated that AM significantly increased the number of apoptotic cells in a time-dependent manner, and l death of HeLa cells by inducing apoptosis.

### AM inhibited proliferation of HeLa cells and arrested the cell cycle at G2/M phase

**Figure 5:**
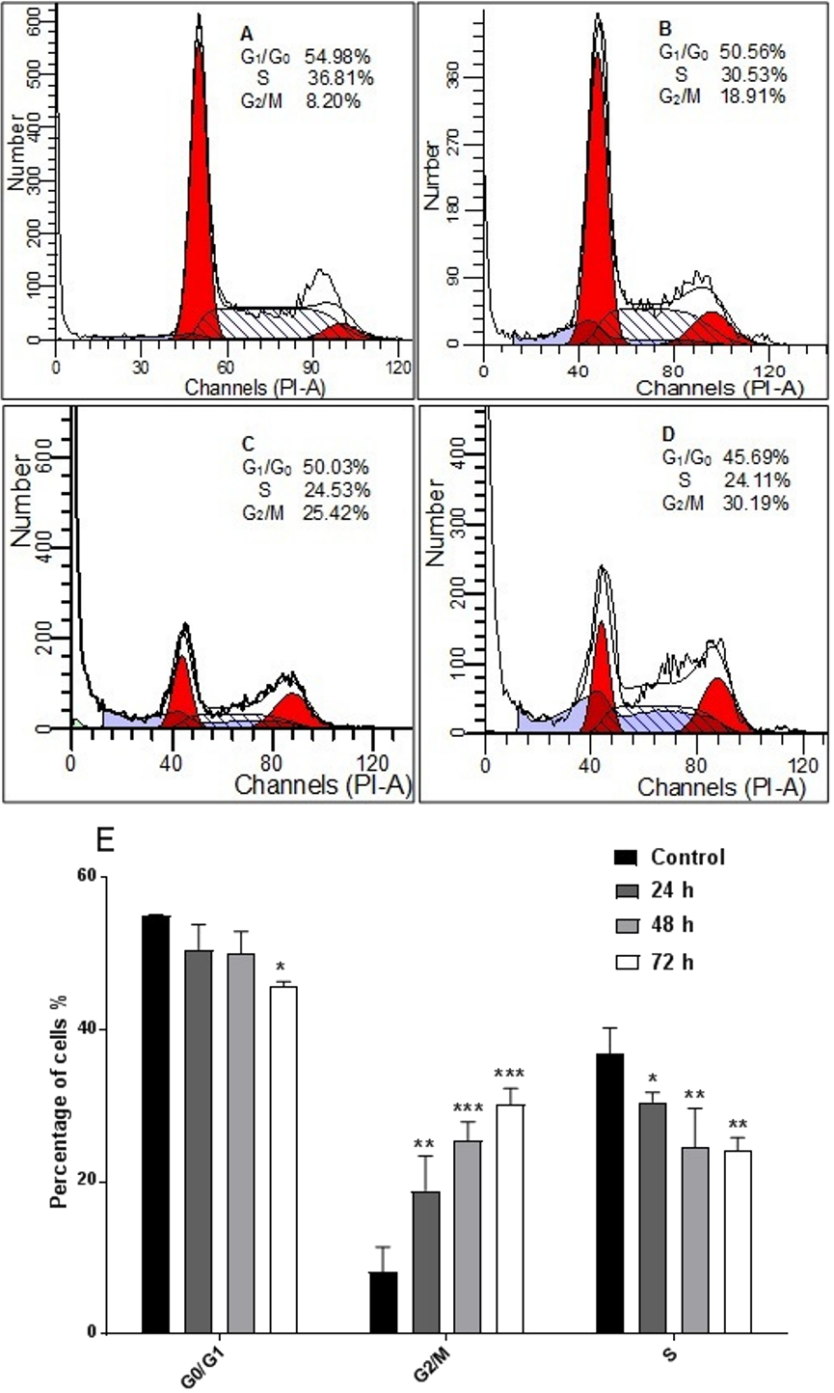
Effects of AM on HeLa cells cell cycle. Histograms of cell cycle flow cytometry analysis for HeLa cells treated with AM (24 µM) for 24 (B), 48 (C) and 72 h (D), where (A) is control. Results represent one of three separated experiments. (E) Promotion of G2/M phase accumulation in the cell cycle progression of HeLa cells by AM, ^∗^*P* < 0.05.

The response of cancer cells to anticancer drugs is affected by the integrity of cell cycle checkpoints (G1 and G2 arrest), which are a potential target of anticancer drugs. The kinetics of the cell cycle was evaluated after 24, 48, and 72 h by flow cytometry analysis. The results demonstrated that AM arrested cell cycle progression in HeLa cells at the G2/M phase in a time-dependent manner (Fig. 5).

### AM induced Caspases activity

**Figure 6:**
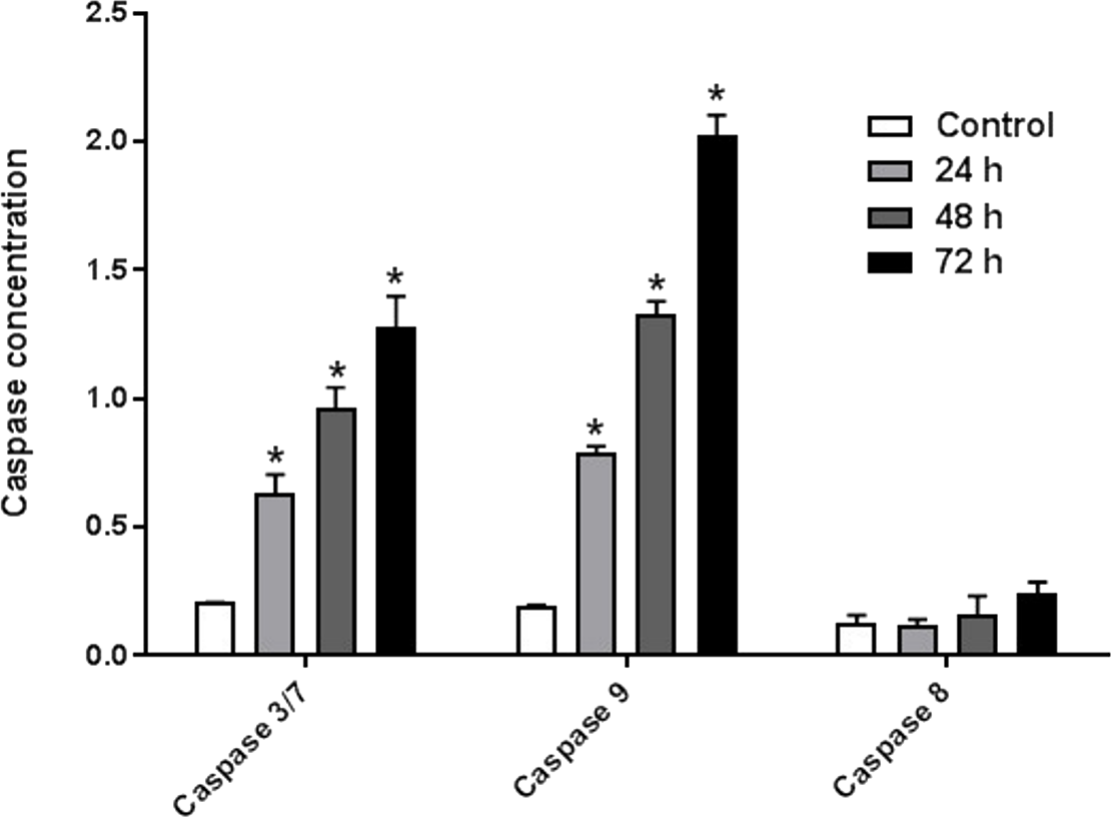
Proportional expressions of Caspases 3/7, 9 and 8 in HeLa cells treated with IC_50_ concentration of AM in time dependent manner. Each treatment group was done in triplicate, ^∗^*P* < 0.05.

The enzyme activities of caspases 3/7, 8, and 9 were measured on HeLa cells treated with IC_50_ of AM for 24, 48, and 72 h. The results indicated that active caspases 3/7 and 9 were present in HeLa cells stimulated with AM in a time-dependent manner. HeLa cells were highly positive to active caspase 3/7 and caspase 9 compared with the control cells (Fig. 6). By contrast, the level of caspase 8 enzyme did not change in HeLa cells after treatment with AM.

### AM increased cellular reactive oxygen species (ROS) level

**Figure 7:**
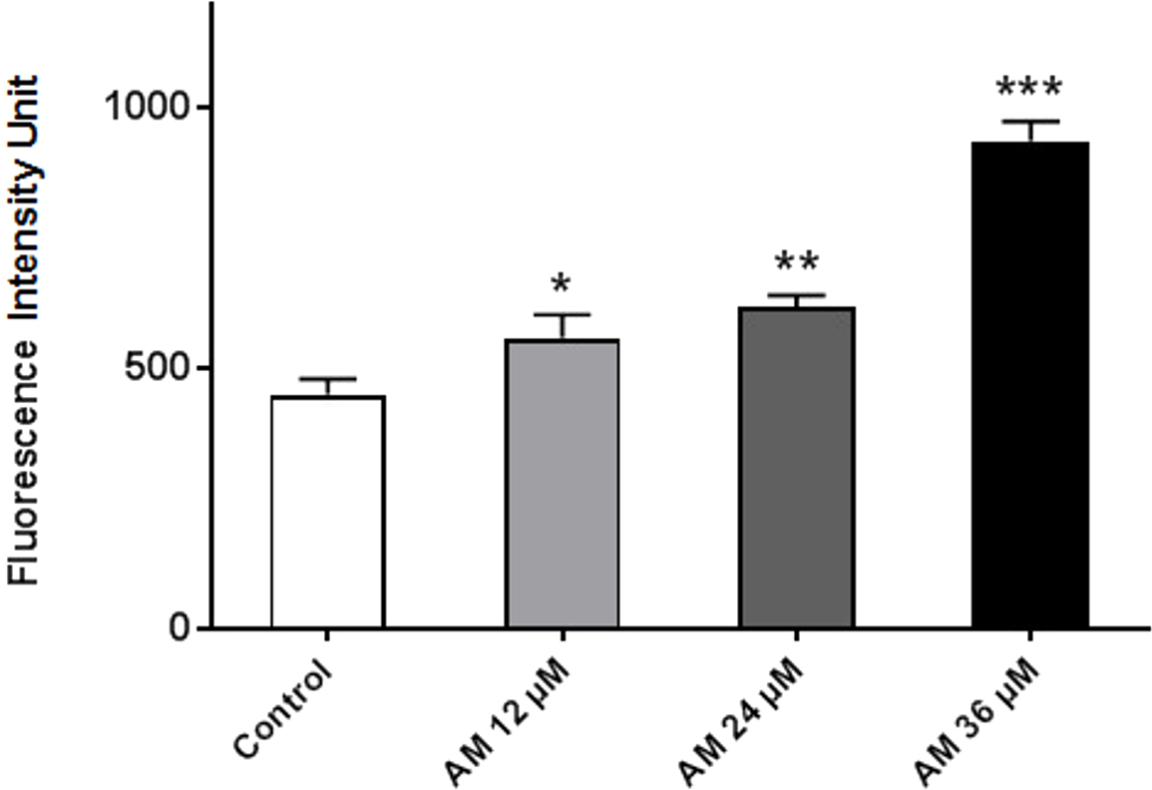
Effect of AM on ROS production in HeLa cells. ROS concentrations were calculated after treating cells with AM (12, 24, and 36 µM). The cells permeant DCFH-DA fluorogenic dye that evaluates ROS production within the cells, and the fluorescence was measured by ELISA microplate reader, ^∗^*P* < 0.05; ^∗∗^*P* < 0.01; ^∗∗∗^*P* < 0.001.

ROS comprises reactive molecules and free radicals containing oxygen molecules, which serve as inter- and intracellular messengers; ROS plays a critical role in cell signaling as apoptosis gene expression and cell signaling cascade activation. These molecules are generated as byproducts during mitochondrial electron transport in aerobic respiration. Studies demonstrated that the role of ROS in cancer is not limited to genetic toxicity and mutation leading to cancer but also as signal transduction messengers. ROS can initiate either cell proliferation or death according to the actual endogenous and exogenous conditions in the cancer cells. As shown in Fig. 7, AM could increase the ROS levels in HeLa cells in a concentration-dependent manner. ROS levels were higher in the treated cells than those in the control cells, and the difference increases with prolonged treatment duration.

### AM induced apoptosis on mitochondrial disruption

**Figure 8:**
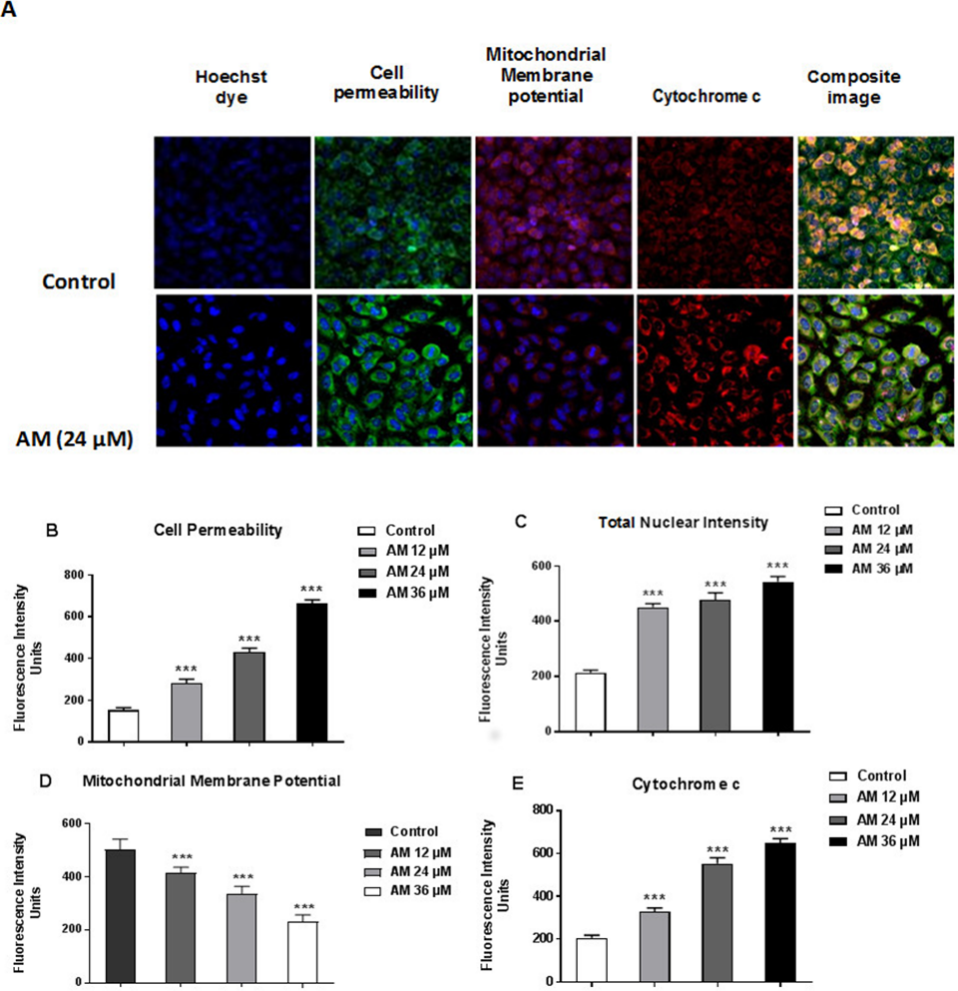
Apoptotic parameters of Hela cells treated with (24 µM) AM. (A) Representative images of HeLa cells treated with AM compared to untreated cells, and stained with Hoechst dye, cell permeability dye, mitochondrial membrane potential dye and cytochrome c. The images of each row are acquired from the same field of the same treatment sample (magnification 20×). Quantitative analysis of changes in (B) Total nuclear intensity, (C) Cell permeability, (D) Mitochondrial membrane potential and (E) Cytochrome c localization were measured simultaneously in HeLa cells. Statistical significance is expressed as ^∗∗^*P* < 0.01; ^∗∗∗^*P* < 0.001.

Cytotoxicity kits measure various parameters related to cell viability, including cells number, nuclear size, morphological changes, cell permeability, mitochondrial membrane potential, and cytochrome c. All cytotoxicity changes were presented in Fig. 8A. The results showed that HeLa cells treated with 24 µM AM exhibited MMP reductions which were indicated by weakened fluorescence intensity. In addition, plasma membrane permeability was increased leading to localization of cytochrome c into cytosol. Data were analyzed using the ArrayScan HCS system (Cellomics, PA, USA). The results showed a significant increase in cells permeability (Fig. 8B), total nuclear intensity (Fig. 8C), and cytochrome c release (Fig. 8E) in treated HeLa cells compared with the control cells in the same graph. Furthermore, it showed a significant decrease in MMP, particularly at high AM concentrations, in treated HeLa cells compared with the control cells as shown in Fig. 8D.

## Discussion

Plants have been used in cancer therapy for thousands of years. Traditional medicine plays a critical role in treatment of many chronic life-threatening conditions and diseases, including cancer (Mohan et al., 2011). The severity of side-effects and acquired resistant of recent chemotherapeutic agents particularly at high doses limit its usage and boost researchers to investigate for other anticancer treatments (Loehrer & Einhorn, 1984; Sitzia & Huggins, 1998). AM is an oxygenated and prenylated xanthone compound isolated from *C. arborescens*, and shows strong pharmacological effects *in vitro* and *in vivo* through various mechanisms of action (Ibrahim et al., 2015; Morelli et al., 2015). In the present study, we attempted to explore the mechanism of action involved in the encouraging effects of AM on human cervical cancer cell line, HeLa. We have previously reported that AM has a suppressive effect on many cancer cell lines including breast cancer MCF-7 cells. This effect was mediated through the induction of an apoptotic process with regulation of NF-κB and Hsp70 protein modulation (Ibrahim et al., 2014b). Also, AM was reported to induce apoptosis via mitochondrial dysfunction in human leukemia HL60 cells (Matsumoto et al., 2004). We used MTT colorimetric assay to assess the viability and proliferation of HeLa cells before and after treatment with AM *in vitro* and then compared the results with the effect of AM on the normal human ovarian cell line SV40. We found that AM exhibited strong cytotoxicity in HeLa cells in a dose- and time-dependent manner with IC_50_ value 24.53 ±  1.48 µM after 24 h as shown in Table 1. AM has no reported toxicological evaluations *in vivo* (Ibrahim et al., 2015), and has low toxicity on normal human epithelial ovarian cells SV40 with IC_50_ value 93.26 ±  3.92 µM after 24 h as shown in Table 1. AM toxic effect can obviously be realized by loss of reproductive integrity and proliferation in HeLa cells. AM was able to significantly inhibit colonies formation of HeLa cells as shown in Fig. 2. This explained that HeLa cells were unable to actively replicate and form colonies after treatment with AM and the inhibition in colony formation increased gradually with increasing in AM concentration. Apoptogenic effect of AM on HeLa has been further illustrated with morphological study utilizing fluorescent microscopy AO/PI staining assay. AO and PI dyes intercalated to DNA fluorochromes therefor, green and orange fluorescence was emitted, respectively. Only AO dye could pass through the viable and early apoptotic cells membranes. Thus, viable cells exhibited green nucleus with normal structure while cells which displayed early apoptosis showed a bright-green nucleus with condensed or fragmented chromatin in the nucleus. Both AO and PI dyes could cross to late apoptotic cells and necrotic cells, so late apoptotic cells exhibited condensed and fragmented orange chromatin; furthermore, secondary necrotic cells exhibited an intense orange nucleus with intact structure as shown in Fig. 3. Apoptosis induced in HeLa cells by AM could be seen obviously by Hoechst 33285 staining, parts of HeLa cells exhibited nuclear condensation which increased gradually with time of exposure to AM until apoptotic bodies, fragmented DNA and dead cells appeared as shown in Fig. 4. The outcome of these results were confirmed by multiparametric cell-based high content screening (HCS) analysis that showed morphological features characteristic of apoptotic cell death such as nuclear condensation, loss of membrane symmetry, release of cytochrome c and reduction in mitochondrial membrane potential.

It is known that the disruption of the mitochondrial membrane potential is an early event in apoptosis and triggers release of cytochrome c and other apoptogenic molecules from the mitochondria to the cytosol (Elmore, 2007; Ly, Grubb & Lawen, 2003). The release of apoptogenic proteins lead to the stimulation of caspases and finally cell death. A lot of molecular reports indicate that the processes of apoptosis can be triggered whether via extrinsic pathway where the ligand-receptor binding triggers caspase-8 or the mitochondrial pathway where cytochrome c is discharged from the mitochondrial (Nair et al., 2014; Vitale et al., 2016). Cytochrome c release to the cytosol controls apoptosome formation (Abdelwahab et al., 2011). Apoptosome activates procaspase-9 to caspase-9 which leads to activation of caspase-3 in the activation cascade of the mitochondrial intrinsic pathway leading to apoptosis (Makowska et al., 2014). The current report revealed that treatment with AM increased activities of caspase-3/-7 and -9 as shown in Fig. 6, and caused collapse of mitochondrial membrane potential (MMP) and discharge of cytochrome c as shown in Figs. 8A and 8B, which leads us to assume that AM can trigger apoptosis via the mitochondrial pathway. Mitochondrial pathway was confirmed by MMP disruption and increase in the cell permeability as shown in Fig. 8A. AM significantly stimulated the cells to release cytochrome c from mitochondria into cytosol during early apoptosis, cytochrome c release to the cytosol stimulates caspase-9, which in turn leads to activation of the downstream caspase-3/7. These results assume that AM induced apoptosis is mediated via the intrinsic or mitochondrial pathway. On the other side, results showed no valuable change in caspase-8 levels in HeLa cells before and after treatment with AM. So that, we can assume that apoptosis in Hela cells is mediated through the intrinsic pathway without the association of extrinsic pathway. Concurrent with the previous events, many studies assume the role of oxidative stress in the process of apoptosis and related mitochondrial alterations (Makowska et al., 2014; Tang et al., 2014). Evidence proves that the effect of intracellular reactive oxygen species (ROS) in cancer is not limited to be cancer promoters that facilitate mutagenesis but also as signal transduction messengers, which may induce either proliferation or death of cancer cells, according to the cellular endogenous and exogenous situations. In order to examine the role of ROS, we assessed the ROS levels upon AM treatment against HeLa. The outcomes obviously highlight this important correlation. Intracellular ROS level increased by almost twofold in HeLa cells treated with 36 µM AM as shown in Fig. 7, and this may be due to the production of free radical during the cytotoxicity. The manner of apoptosis initiated by a lot of natural agents is intimately associated to cell cycle halt (Shoja et al., 2015). It has been documented that cell cycle control is a substantial incident in protection precise cellular division. Numerous carcinogenic progressions are reported to produce the defects of cell cycle regulators. Therefore, it is a rational goal to change the cell cycle regulators in cancer cells for the objective of chemoprevention and chemotherapy (Saleh et al., 2015; Yang et al., 2015). After treatment with AM for 24, 48 and 72 h, the investigation of the cell cycle of HeLa cells revealed a higher percentage of cells in the G2/M phase. In contrast, the percentage of cells in the S and G0/G1 phase decreased in comparison with the untreated cells as shown in Fig. 5. These outcomes show the ability of AM to inhibit cellular proliferation through G2/M phase arrest.

## Conclusions

In conclusion, in this report we give demonstration that AM induces apoptosis in human cervical cancer cells (HeLa), through the intrinsic pathway and promotion of intracellular reactive oxygen species production. In addition, AM is capable to induce HeLa cell arrest at G2/M phase and adjust the MMP to trigger cytochrome c release from the mitochondria to the cytosol which activates caspases series leading to apoptosis. Our findings may assist to a better illustration of the apoptotic mechanisms through which AM exerts its effects towards cervical cancer cells (HeLa) and may lead to the development of new chemotherapeutic treatment for the cure of cervical cancer, for which there is no efficient life-prolonging treatments.

##  Supplemental Information

10.7717/peerj.3460/supp-1 Table S1 Raw data—Table 1

10.7717/peerj.3460/supp-2 Table S2 Raw data—Table 2

10.7717/peerj.3460/supp-3 Table S3 Raw data—Table 3

10.7717/peerj.3460/supp-4 Table S4 Raw data—Table 4

10.7717/peerj.3460/supp-5 Table S5 Raw data—Table 5

10.7717/peerj.3460/supp-6 Table S6 Raw data—Table 6

10.7717/peerj.3460/supp-7 Table S7 Raw data—Table 7

## Additional Information and Declarations

### Data Availability

The following information was supplied regarding data availability: The raw data has been supplied as a Supplementary File.
