# Supplementary material for: In vitro assessment of anti-proliferative effect induced by α-mangostin from Cratoxylum arborescens on HeLa cells
Source: PeerJ. 2017 Jul 21;5:e3460. doi: 10.7717/peerj.3460 (PMC5522721; doi:10.7717/peerj.3460)
Supplement: Table S1 [file peerj-05-3460-s001.docx]

**Raw Data for MTT Test**

1. **MTT by AM on HeLa cells**

**For 24 hour**

Experiment 1: IC_50_ = 10.55

| column 1 | column 2 | column 3 | average |
| --- | --- | --- | --- |
| 0.1654 | 0.142 | 0.1072 | 0.1382 |
| 0.2403 | 0.2842 | 0.2202 | 0.248233 |
| 0.2454 | 0.3317 | 0.2331 | 0.270067 |
| 0.3947 | 0.4149 | 0.3872 | 0.398933 |
| 0.5295 | 0.5218 | 0.5068 | 0.519367 |
| 0.7284 | 0.7289 | 0.6967 | 0.718 |
| 0.8716 | 0.8928 | 0.8599 | 0.874767 |
| 0.9827 | 0.9091 | 0.9228 | 0.9382 |

Experiment 2: IC_50_ = 9.02

| column 1 | column 2 | column 3 | average |
| --- | --- | --- | --- |
| 0.2022 | 0.2031 | 0.2027 | 0.202667 |
| 0.2279 | 0.2472 | 0.2266 | 0.2339 |
| 0.2522 | 0.2358 | 0.2782 | 0.2554 |
| 0.2758 | 0.2776 | 0.2716 | 0.275 |
| 0.6065 | 0.6056 | 0.5996 | 0.6039 |
| 0.99447 | 0.8917 | 0.948 | 0.944723 |
| 0.9649 | 0.9572 | 1.0934 | 1.005167 |
| 1.1044 | 1.1118 | 1.0327 | 1.082967 |

Experiment 3: IC_50_ = 10.46

| column 1 | column 2 | column 3 | average |
| --- | --- | --- | --- |
| 0.2067 | 0.1819 | 0.1959 | 0.194833 |
| 0.2195 | 0.2024 | 0.1985 | 0.2068 |
| 0.2345 | 0.2393 | 0.2237 | 0.2325 |
| 0.3496 | 0.3112 | 0.3517 | 0.3375 |
| 0.578 | 0.583 | 0.5819 | 0.580967 |
| 0.7883 | 0.8053 | 0.7966 | 0.796733 |
| 0.8586 | 0.8489 | 0.8388 | 0.848767 |
| 0.9559 | 0.903 | 0.9324 | 0.930433 |

Mean IC_50_ = 10.07

SD IC_50_ = 0.61

**For 48 hour**

Experiment 1 IC_50_ = 6.87

| column 1 | column 2 | column 3 | Average |
| --- | --- | --- | --- |
| 0.0967 | 0.08419 | 0.0927 | 0.091197 |
| 0.1195 | 0.1234 | 0.1143 | 0.119067 |
| 0.1541 | 0.1593 | 0.1637 | 0.159033 |
| 0.2091 | 0.2202 | 0.2017 | 0.210333 |
| 0.4478 | 0.453 | 0.4419 | 0.447567 |
| 0.6883 | 0.6053 | 0.6966 | 0.6634 |
| 0.8586 | 0.8489 | 0.8388 | 0.848767 |
| 1.1559 | 0.903 | 0.9324 | 0.9971 |

Experiment 2: IC_50_ = 4.55

| column 1 | column 2 | column 3 | Average |
| --- | --- | --- | --- |
| 0.0921 | 0.0512 | 0.0727 | 0.072 |
| 0.1309 | 0.1072 | 0.1166 | 0.118233 |
| 0.1622 | 0.1658 | 0.1382 | 0.1554 |
| 0.2158 | 0.2076 | 0.1956 | 0.206333 |
| 0.3065 | 0.3056 | 0.3296 | 0.3139 |
| 0.4947 | 0.5017 | 0.5048 | 0.5004 |
| 0.8049 | 0.7872 | 0.7934 | 0.795167 |
| 0.9601 | 0.9651 | 1.0809 | 1.002033 |

Experiment 3: IC_50_ = 5.93

| column 1 | column 2 | column 3 | Average |
| --- | --- | --- | --- |
| 0.0654 | 0.1042 | 0.0772 | 0.082267 |
| 0.1403 | 0.142 | 0.1202 | 0.134167 |
| 0.1952 | 0.1917 | 0.1731 | 0.186667 |
| 0.2347 | 0.2245 | 0.2272 | 0.2288 |
| 0.4295 | 0.4218 | 0.3068 | 0.386033 |
| 0.5267 | 0.5243 | 0.5061 | 0.519033 |
| 0.716 | 0.7534 | 0.7687 | 0.746033 |
| 0.9827 | 0.9091 | 0.9228 | 0.9382 |

Mean IC_50_ = 5.78

SD IC_50_ = 0.95

**For 72 Hour**

Experiment 1 IC_50_ = 5.11

| column 1 | column 2 | column 3 | average |
| --- | --- | --- | --- |
| 0.067 | 0.1012 | 0.0411 | 0.069767 |
| 0.1195 | 0.1024 | 0.0903 | 0.104067 |
| 0.1345 | 0.1293 | 0.1122 | 0.125333 |
| 0.1856 | 0.1732 | 0.1617 | 0.1735 |
| 0.2978 | 0.2683 | 0.3101 | 0.292067 |
| 0.5883 | 0.6053 | 0.6634 | 0.619 |
| 0.9586 | 0.8489 | 0.7388 | 0.848767 |
| 1.0059 | 1.0903 | 1.0024 | 1.032867 |

Experiment 2: IC_50_ = 4.61

| column 1 | column 2 | column 3 | average |
| --- | --- | --- | --- |
| 0.0822 | 0.0731 | 0.0727 | 0.076 |
| 0.1001 | 0.0972 | 0.1066 | 0.1013 |
| 0.1501 | 0.1756 | 0.1302 | 0.151967 |
| 0.2008 | 0.2076 | 0.2016 | 0.203333 |
| 0.2999 | 0.286 | 0.2696 | 0.285167 |
| 0.69447 | 0.607 | 0.5948 | 0.63209 |
| 0.9649 | 0.8572 | 1.023 | 0.948367 |
| 1.1623 | 1.1822 | 1.1623 | 1.168933 |

Experiment 3: IC_50_ = 5.92

| column 1 | column 2 | column 3 | Average |
| --- | --- | --- | --- |
| 0.0654 | 0.1042 | 0.1072 | 0.092267 |
| 0.1403 | 0.1042 | 0.1234 | 0.122633 |
| 0.1989 | 0.2066 | 0.1099 | 0.1718 |
| 0.2047 | 0.2149 | 0.2045 | 0.208033 |
| 0.2395 | 0.2218 | 0.3061 | 0.2558 |
| 0.6284 | 0.5289 | 0.6967 | 0.618 |
| 0.8716 | 0.8028 | 0.8599 | 0.844767 |
| 0.9944 | 0.9676 | 0.9228 | 0.9616 |

Mean IC_50_ = 5.21

SD IC_50_ = 0.53

1. **MTT by AM on HeLa cells**

**For 24 hour**

Experiment 1: IC_50_ = 37.41

| column 1 | column 2 | column 3 | average |
| --- | --- | --- | --- |
| 0.1735 | 0.1957 | 0.1831 | 0.1841 |
| 0.2387 | 0.275 | 0.2731 | 0.262267 |
| 0.4582 | 0.4916 | 0.4907 | 0.480167 |
| 0.5775 | 0.5996 | 0.6273 | 0.601467 |
| 0.5999 | 0.6984 | 0.6533 | 0.650533 |
| 0.6583 | 0.6525 | 0.6586 | 0.656467 |
| 0.7216 | 0.6429 | 0.7504 | 0.704967 |
| 0.7571 | 0.6021 | 0.7643 | 0.707833 |

Experiment 1: IC_50_ = 36.88

| column 1 | column 2 | column 3 | average |
| --- | --- | --- | --- |
| 0.1735 | 0.1667 | 0.1666 | 0.168933 |
| 0.3545 | 0.3275 | 0.3731 | 0.3517 |
| 0.4121 | 0.4916 | 0.5907 | 0.498133 |
| 0.5775 | 0.5675 | 0.6567 | 0.600567 |
| 0.5945 | 0.5584 | 0.6565 | 0.603133 |
| 0.6583 | 0.6544 | 0.6586 | 0.6571 |
| 0.7216 | 0.7429 | 0.7504 | 0.7383 |
| 0.8571 | 0.8021 | 0.8643 | 0.841167 |

Experiment 1: IC50 = 40.54

| column 1 | column 2 | column 3 | Average |
| --- | --- | --- | --- |
| 0.1735 | 0.1957 | 0.1831 | 0.1841 |
| 0.4397 | 0.4375 | 0.4331 | 0.436767 |
| 0.4982 | 0.5234 | 0.5055 | 0.509033 |
| 0.5987 | 0.5944 | 0.6073 | 0.600133 |
| 0.6933 | 0.6984 | 0.693 | 0.6949 |
| 0.6773 | 0.6529 | 0.7116 | 0.6806 |
| 0.8986 | 0.8654 | 0.9504 | 0.9048 |
| 0.7571 | 0.9021 | 0.8643 | 0.841167 |

MeanIC_50_ = 38.28

SD IC_50_ = 1.61
