# Supplementary material for: In vitro assessment of anti-proliferative effect induced by α-mangostin from Cratoxylum arborescens on HeLa cells
Source: PeerJ. 2017 Jul 21;5:e3460. doi: 10.7717/peerj.3460 (PMC5522721; doi:10.7717/peerj.3460)
Supplement: Table S2 [file peerj-05-3460-s002.docx]

**Raw Data for Clonogenic Assay:**

Experiment 1

| Drug Conc. | No. of cell plated | No. of colonies counted | plating efficiency |
| --- | --- | --- | --- |
| CONTROL | 400 | 600 | 150 |
| 5 | 400 | 155 | 38.75 |
| 10 | 400 | 50 | 12.5 |
| 15 | 400 | 24 | 6 |

Experiment 2

| Drug Conc. | No. of cell plated | No. of colonies counted | plating efficiency |
| --- | --- | --- | --- |
| CONTROL | 400 | 570 | 142.5 |
| 5 | 400 | 100 | 25 |
| 10 | 400 | 75 | 18.75 |
| 15 | 400 | 40 | 10 |

Experiment 3

| Drug Conc. | No. of cell plated | No. of colonies counted | plating efficiency |
| --- | --- | --- | --- |
| CONTROL | 400 | 630 | 157.5 |
| 5 | 400 | 120 | 30 |
| 10 | 400 | 85 | 21.25 |
| 15 | 400 | 35 | 8.75 |

| Survival fraction Experiment 1 | Survival fraction Experiment 2 | Survival fraction Experiment 3 | Mean | SD |
| --- | --- | --- | --- | --- |
| 100 | 100 | 100 | 100 | 0 |
| 25.84 | 17.54 | 19.04 | 20.80667 | 3.611402 |
| 8.34 | 13.15 | 13.49 | 11.66 | 2.351694 |
| 4 | 6.67 | 5.56 | 5.41 | 1.095171 |
