# Supplementary material for: In vitro assessment of anti-proliferative effect induced by α-mangostin from Cratoxylum arborescens on HeLa cells
Source: PeerJ. 2017 Jul 21;5:e3460. doi: 10.7717/peerj.3460 (PMC5522721; doi:10.7717/peerj.3460)
Supplement: Table S3 [file peerj-05-3460-s003.docx]

**Raw Data for AOPI Assay**

Experiment 1:

|  | V% | EA% | LA% | N% |
| --- | --- | --- | --- | --- |
| control | 85 | 10 | 3 | 2 |
| 24 | 50 | 25 | 15 | 10 |
| 48 | 25 | 35 | 25 | 15 |
| 72 | 10 | 40 | 34 | 16 |

Experiment 2:

|  | V% | EA% | LA% | N% |
| --- | --- | --- | --- | --- |
| control | 80 | 12 | 5 | 3 |
| 24 | 50 | 25 | 17 | 8 |
| 48 | 28 | 35 | 25 | 12 |
| 72 | 14 | 40 | 33 | 13 |

Experiment 3:

|  | V% | EA% | LA% | N% |
| --- | --- | --- | --- | --- |
| control | 78 | 12 | 4 | 5 |
| 24 | 44 | 28 | 16 | 12 |
| 48 | 26 | 34 | 25 | 15 |
| 72 | 11 | 40 | 33 | 16 |

Mean

|  | V% | EA% | LA% | N% |
| --- | --- | --- | --- | --- |
| 0 | 81 | 12 | 4 | 4 |
| 24 | 48 | 26 | 16 | 10 |
| 48 | 26.33333 | 33.33333 | 25 | 14 |
| 72 | 11.66667 | 40 | 33.33333 | 15 |

SD

|  | V | EA | LA | N |
| --- | --- | --- | --- | --- |
| 0 | 2.94392 | 0 | 0.816497 | 0.816497 |
| 24 | 2.828427 | 1.414214 | 0.816497 | 1.632993 |
| 48 | 1.247219 | 0.471405 | 0 | 1.414214 |
| 72 | 1.699673 | 0 | 0.471405 | 1.414214 |
