# Supplementary material for: In vitro assessment of anti-proliferative effect induced by α-mangostin from Cratoxylum arborescens on HeLa cells
Source: PeerJ. 2017 Jul 21;5:e3460. doi: 10.7717/peerj.3460 (PMC5522721; doi:10.7717/peerj.3460)
Supplement: Table S4 [file peerj-05-3460-s004.docx]

**Raw Data for Cell Cycle Assay**

Experiment 1:

|  | G0/G1% | G2/M% | S% |
| --- | --- | --- | --- |
| control | 54.83 | 5.92 | 39.25 |
| 24 h | 47.97 | 22.24 | 29.79 |
| 48 h | 53.17 | 26.61 | 20.21 |
| 72 h | 46.08 | 31.33 | 22.59 |

Experiment 2:

|  | G0/G1% | G2/M% | S% |
| --- | --- | --- | --- |
| control | 54.96 | 5.84 | 39.21 |
| 24 h | 48.56 | 21.94 | 29.5 |
| 48 h | 50.7 | 27.71 | 21.59 |
| 72 h | 44.75 | 32.03 | 23.21 |

Experiment 3:

|  | G0/G1% | G2/M% | S% |
| --- | --- | --- | --- |
| control | 55.16 | 12.84 | 31.99 |
| 24 h | 55.15 | 12.55 | 32.3 |
| 48 h | 46.24 | 21.96 | 31.8 |
| 72 h | 46.24 | 27.23 | 26.53 |

Mean

|  | G0/G1% | G2/M% | S% |
| --- | --- | --- | --- |
| control | 54.98333 | 8.20 | 36.81667 |
| 24 h | 50.56 | 18.91 | 30.53 |
| 48 h | 50.03667 | 25.42667 | 24.53333 |
| 72 h | 45.69 | 30.19667 | 24.11 |

SD

|  | G0/G1% | G2/M% | S% |
| --- | --- | --- | --- |
| control | 0.135728 | 3.281138 | 3.413008 |
| 24 h | 3.254546 | 4.498867 | 1.257166 |
| 48 h | 2.867779 | 2.492099 | 5.169103 |
| 72 h | 0.667882 | 2.117126 | 1.729817 |
