# Supplementary material for: In vitro assessment of anti-proliferative effect induced by α-mangostin from Cratoxylum arborescens on HeLa cells
Source: PeerJ. 2017 Jul 21;5:e3460. doi: 10.7717/peerj.3460 (PMC5522721; doi:10.7717/peerj.3460)
Supplement: Table S5 [file peerj-05-3460-s005.docx]

**Raw Data for Caspases**

1. **Caspase 3:**

| time | Experiment 1 | Experiment 2 | Experiment 3 |
| --- | --- | --- | --- |
| 0 | 0.2043 | 0.2086 | 0.2073 |
| 24 | 0.7333 | 0.5647 | 0.5941 |
| 48 | 1.0038 | 0.8604 | 1.0359 |
| 72 | 1.1209 | 1.4131 | 1.3043 |

Mean and SD

| time | mean | SD |
| --- | --- | --- |
| 0 | 0.206733 | 0.001801 |
| 24 | 0.6307 | 0.073535 |
| 48 | 0.9667 | 0.076299 |
| 72 | 1.279433 | 0.120579 |

1. **Caspase 8: (not active)**

| time | Experiment 1 | Experiment 2 | Experiment 3 |
| --- | --- | --- | --- |
| 0 | 0.1273 | 0.1096 | 0.1258 |
| 24 | 0.166 | 0.1654 | 0.1543 |
| 48 | 0.1966 | 0.1811 | 0.1879 |
| 72 | 0.2412 | 0.2477 | 0.2542 |

Mean and SD (not active)

| time | mean | SD |
| --- | --- | --- |
| 0 | 0.120872 | 0.008019 |
| 24 | 0.161922 | 0.005375 |
| 48 | 0.188523 | 0.006321 |
| 72 | 0.247698 | 0.005306 |

1. **Caspase 9:**

| time | Experiment 1 | Experiment 2 | Experiment 3 |
| --- | --- | --- | --- |
| 0 | 0.1975 | 0.1907 | 0.1916 |
| 24 | 0.7603 | 0.8216 | 0.7922 |
| 48 | 1.2608 | 1.3376 | 1.3863 |
| 72 | 1.9565 | 2.1377 | 1.9727 |

Mean and SD

| time | mean | SD |
| --- | --- | --- |
| 0 | 0.193267 | 0.003015884 |
| 24 | 0.791367 | 0.025032557 |
| 48 | 1.328233 | 0.051661484 |
| 72 | 2.0223 | 0.081867698 |
