# Supplementary material for: In vitro assessment of anti-proliferative effect induced by α-mangostin from Cratoxylum arborescens on HeLa cells
Source: PeerJ. 2017 Jul 21;5:e3460. doi: 10.7717/peerj.3460 (PMC5522721; doi:10.7717/peerj.3460)
Supplement: Table S6 [file peerj-05-3460-s006.docx]

**Raw Data for ROS Production:**

| AM conc. | Experiment 1 | Experiment 2 | Experiment 3 |
| --- | --- | --- | --- |
| 0 μg/mL | 460 | 474 | 418 |
| 5 μg/mL | 508 | 578 | 591 |
| 10 μg/mL | 601 | 615 | 642 |
| 15 μg/mL | 960 | 894 | 957 |

Mean and SD

| AM conc. | mean | SD |
| --- | --- | --- |
| 0 μg/mL | 450.6667 | 23.79542 |
| 5 μg/mL | 559 | 36.45088 |
| 10 μg/mL | 619.3333 | 17.01633 |
| 15 μg/mL | 937 | 30.43025 |
