# Supplementary material for: In vitro assessment of anti-proliferative effect induced by α-mangostin from Cratoxylum arborescens on HeLa cells
Source: PeerJ. 2017 Jul 21;5:e3460. doi: 10.7717/peerj.3460 (PMC5522721; doi:10.7717/peerj.3460)
Supplement: Table S7 [file peerj-05-3460-s007.docx]

**Raw Data for cytotoxicity 3 kit:**

1. **Total Nuclear Intensity**

| AM conc. | Experiment 1 | Experiment 2 | Experiment 3 |
| --- | --- | --- | --- |
| 0 μg/mL | 233.36 | 225.77 | 214.15 |
| 5 μg/mL | 413.71 | 433.41 | 417.78 |
| 10 μg/mL | 474.96 | 457.35 | 417.24 |
| 15 μg/mL | 523.31 | 542.81 | 515.81 |

Mean and SD

| AM conc. | mean | SD |
| --- | --- | --- |
| 0 μg/mL | 224.4267 | 7.899765 |
| 5 μg/mL | 421.6333 | 8.491511 |
| 10 μg/mL | 449.85 | 24.1535 |
| 15 μg/mL | 527.31 | 11.37981 |

1. **Mitochondrial Membrane Potential**

| AM conc. | Experiment 1 | Experiment 2 | Experiment 3 |
| --- | --- | --- | --- |
| 0 μg/mL | 515.45 | 544.11 | 475.44 |
| 5 μg/mL | 411.23 | 385.12 | 428.19 |
| 10 μg/mL | 325.77 | 380.56 | 333.77 |
| 15 μg/mL | 222.76 | 239.21 | 192.56 |

Mean and SD

| AM conc. | mean | SD |
| --- | --- | --- |
| 0 μg/mL | 511.6667 | 28.16176 |
| 5 μg/mL | 408.18 | 17.71502 |
| 10 μg/mL | 346.7 | 24.16436 |
| 15 μg/mL | 218.1767 | 19.31857 |

1. **Cell Permeability**

| AM conc. | Experiment 1 | Experiment 2 | Experiment 3 |
| --- | --- | --- | --- |
| 0 μg/mL | 167.36 | 188.77 | 172.15 |
| 5 μg/mL | 283.71 | 253.41 | 317.78 |
| 10 μg/mL | 455.96 | 412.35 | 413.44 |
| 15 μg/mL | 633.32 | 602.22 | 615.65 |

Mean and SD

| AM conc. | mean | SD |
| --- | --- | --- |
| 0 μg/mL | 176.0933 | 9.174582 |
| 5 μg/mL | 284.9667 | 26.29396 |
| 10 μg/mL | 427.25 | 20.30591 |
| 15 μg/mL | 617.0633 | 12.73579 |

1. **Cytochrome C**

| AM conc. | Experiment 1 | Experiment 2 | Experiment 3 |
| --- | --- | --- | --- |
| 0 μg/mL | 214.45 | 212.11 | 202.34 |
| 5 μg/mL | 321.43 | 325.16 | 289.19 |
| 10 μg/mL | 573.33 | 530.56 | 598.53 |
| 15 μg/mL | 622.33 | 619.41 | 675.56 |

Mean and SD

| AM conc. | Mean | SD |
| --- | --- | --- |
| 0 μg/mL | 209.6333 | 5.244898 |
| 5 μg/mL | 311.9267 | 16.14921 |
| 10 μg/mL | 567.4733 | 28.05596 |
| 15 μg/mL | 639.1 | 25.80866 |
